# A Novel Black Widow Optimization Algorithm Based on Lagrange Interpolation Operator for ResNet18

**DOI:** 10.3390/biomimetics10060361

**Published:** 2025-06-03

**Authors:** Peiyang Wei, Can Hu, Jingyi Hu, Zhibin Li, Wen Qin, Jianhong Gan, Tinghui Chen, Hongping Shu, Mingsheng Shang

**Affiliations:** 1 School of Computer Science and Technology, Chongqing University of Posts and Telecommunications, Chongqing 400065, China; chenth199208@outlook.com; 2 School of Software Engineering, Chengdu University of Information Technology, Chengdu 610225, China; hucan028@outlook.com (C.H.); 2645480469hjy@gmail.com (J.H.); lizhibin@cuit.edu.cn (Z.L.); gjh@cuit.edu.cn (J.G.); shp@cuit.edu.cn (H.S.); 3 Chongqing Institute of Green and Intelligent Technology, Chinese Academy of Sciences, Chongqing 400714, China; msshang@cigit.ac.cn; 4 Automatic Software Generation & Intelligence Service Key Laboratory of Sichuan Province, Chengdu 610225, China; 5 Key Laboratory of Remote Sensing Application and Innovation, Chongqing 401147, China; 6 School of Computer Science, Sichuan Normal University, Chengdu 610101, China; qinwen@sicnu.edu.cn

**Keywords:** ResNet18, adaptive learning rate, black widow optimization algorithm, Lagrange interpolation

## Abstract

Hyper-parameters play a critical role in neural networks; they significantly impact both training effectiveness and overall model performance. Proper hyper-parameter settings can accelerate model convergence and improve generalization. Among various hyper-parameters, the learning rate is particularly important. However, optimizing the learning rate typically requires extensive experimentation and tuning, as its setting is often dependent on specific tasks and datasets and therefore lacks universal rules or standards. Consequently, adjustments are generally made through trial and error, thereby making the selection of the learning rate complex and time-consuming. In an attempt to surmount this challenge, evolutionary computation algorithms can automatically adjust the hyper-parameter learning rate to improve training efficiency and model performance. In response to this, we propose a black widow optimization algorithm based on Lagrange interpolation (LIBWONN) to optimize the learning rate of ResNet18. Moreover, we evaluate LIBWONN’s effectiveness using 24 benchmark functions from CEC2017 and CEC2022 and compare it with nine advanced metaheuristic algorithms. The experimental results indicate that LIBWONN outperforms the other algorithms in convergence and stability. Additionally, experiments on publicly available datasets from six different fields demonstrate that LIBWONN improves the accuracy on both training and testing sets compared to the standard BWO, with gains of 6.99% and 4.48%, respectively.

## 1. Introduction

Owing to its ability to efficiently extract features, scale well, and perform excellently, image recognition technology finds widespread use in fields such as image classification and medical image analysis [1,2,3,4,5]. ResNet, proposed by He et al. [2], addresses the degradation problem in deep neural networks by adding shortcut connections. This design enables the network to be deeper without easily overfitting. This model performs excellently in tasks such as image classification [3], which has become a significant benchmark in deep learning [6,7,8,9,10,11].

The training process of ResNet is significantly affected by its learning rate [4], which determines the step size for updating model parameters and directly influences the convergence speed [12,13,14,15,16], ultimately impacting the model’s performance. Compared to a fixed learning rate, an adaptive learning rate can dynamically adjust based on the model’s performance. This approach effectively addresses complex optimization problems, thereby improving the stability and efficiency of model training [5].

The learning rate determines the step size for updating model parameters, affecting both convergence speed and final performance. It may lead to slow convergence or suffer from a local optimum in a model with improper parameters, which makes it difficult to find a global optimum. Numerous researchers have conducted extensive studies to optimize the learning rate, improving training effectiveness [17,18,19,20,21].

Ma et al. propose an efficient optimization method to address function approximation problems, enabling the solution of partial differential equations with deep learning. They employ particle methods (PMs) and smoothed particle methods (SPMs) for spatial discretization, allowing for a smaller learning rate to ensure the convergence of the optimization algorithm [8]. Franchini et al. study a stochastic gradient algorithm that gradually increases the mini-batch size in a predefined manner, automatically adjusting the learning rate in a line search process that is either monotonic or non-monotonic [9]. Wang et al. adopt a learning rate scheduler with an incremental proportional–integral–derivative controller for optimizing the parameters of stochastic gradient descent (SGD) [10]. Qin et al. developed the Adaptive Parallel Stochastic Gradient Descent (AP-SGD) algorithm to minimize scheduling costs. This method achieved significant parallelism by integrating an adaptive momentum technique into the learning process, thereby speeding up convergence with adaptive learning rates and acceleration coefficients [11]. However, these methods still suffer from inappropriate hyper-parameters, which result in low computational efficiency [22,23,24,25,26,27].

To address the aforementioned issues, this study proposes a Lagrange interpolation black widow optimization algorithm (LIBWONN) based on Lagrange interpolation to further enhance its performance. By dynamically constructing an interpolation function with known data points during iteration, the parameters are adjusted to obtain an optimal learning rate.

In order to substantiate the efficacy of the algorithm proposed herein, nine optimization algorithms are deliberately selected as baselines, which comprehensively embrace four aspects. These aspects include “Optimization based on principles from physics and mathematics (PSEQADE [28])”, “Optimization based on evolutionary cycles (COVIDOA [29], LSHADE-cnEpSin [30])”, “Optimization based on behaviors observed in animals and plants (ALA [31], SDO [32], SASS [33], BOA [34], WOA [35])”, and “Optimization inspired by human activities (DOA [36], CBSO [37], AGSK [38])”. Each of these algorithms represents a different approach to solving optimization problems, ensuring a comprehensive comparison. We conduct comparative experiments to analyze their performance in terms of convergence speed, solution accuracy, and robustness. By comparing these algorithms, we aim to highlight the strengths and weaknesses of LIBWONN.

The key contributions of this study are listed below:

(a) This paper proposes a Lagrange interpolation-based black widow optimization algorithm (LIBWONN) to improve training efficiency and performance by optimizing the learning rate of ResNet18, which overcomes the limitations of the original BWO algorithm in avoiding local optima, achieving higher robust learning rate adjustments.

(b) Experiments are conducted on six publicly available datasets, with nine novel metaheuristic optimization algorithms selected as baselines. The experimental results demonstrate that LIBWONN outperforms the other algorithms and maintains good generalization and stability across multiple datasets.

## 2. Related Theoretical Description

### 2.1. ResNet18 Model

ResNet is a deep residual network model proposed by a research team at Microsoft Research. The number of convolutional layers in ResNet can be adjusted based on different tasks; adding more layers can improve accuracy to meet varying task requirements by increasing structural complexity with depth [38,39,40,41,42,43,44,45,46]. This paper adopts ResNet18, which is a commonly used ResNet model. The primary innovation of this model is the introduction of residual learning. Traditional deep neural networks frequently face issues with vanishing or exploding gradients, especially as the network depth increases, which makes training challenging. ResNet18 addresses these issues by using residual blocks, which allow shortcut connections between network layers, thus enabling the model to learn features more deeply and effectively. This can be represented by the formula below:

(1) H(x)=F(x)+x,

where *H*(*x*) is the expected output of the network, *F*(*x*) denotes the residual function, and *x* is the input signal or feature map.

**Figure 1:**
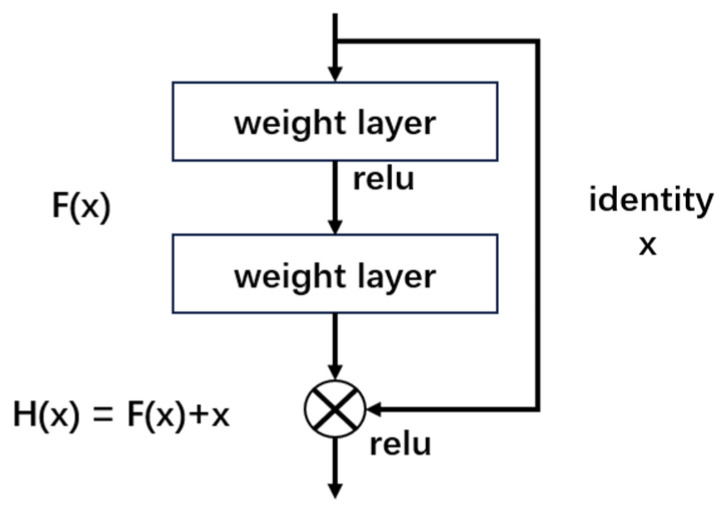
The residual block for ResNet18.

**Figure 2:**
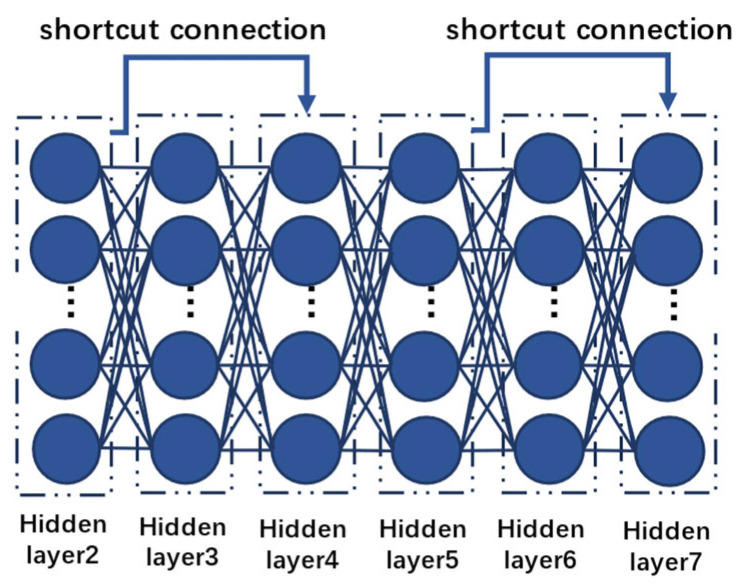
The convolutional image of a residual block.

From Figure 1, each residual block includes two main paths: one is direct identity mapping, where *x* is added directly to the output of the target function via a shortcut connection, as shown in Figure 2; the other is the path for learning the residual, which produces an output through two convolutional layers and the activation function ReLU.

**Figure 3:**
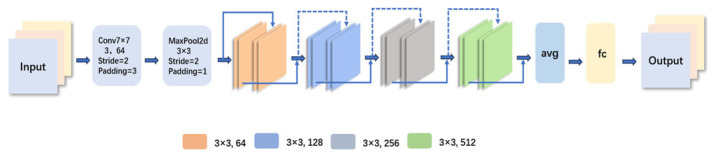
The model architecture diagram of ResNet.

The structure of ResNet18 consists of several major components, and Figure 3 represents the architecture of this model. The input layer accepts an image of size 224 × 224 × 3. This is followed by a 7 × 7 convolutional layer with a stride of 2, outputting 64 channels, and then a 3 × 3 max pooling layer with a stride of 2. In the residual block, the network is divided into four stages. Stage 1 contains two residual blocks with 64 channels; Stage 2 has two residual blocks with 128 channels and a stride of 2; Stages 3 and 4 contain two residual blocks with 256 and 512 channels, respectively, each with a stride of 2. After a global average pooling layer, the feature map is converted into a fixed-length feature vector, which is passed through a fully connected layer to produce a classification result [35,36,37,38,39,40,41].

The learning rate is a critical hyper-parameter for training ResNet18, as it directly affects the convergence speed and performance of the model. An appropriate learning rate can accelerate convergence, allowing the model to achieve better results within fewer training iterations. Setting the learning rate too high can cause the model to overshoot the optimal solution during loss function optimization, resulting in unstable or divergent training. Conversely, if the learning rate is too low, convergence may be slow, increasing training time and potentially trapping the model into local optima. For ResNet18, the depth structure with shortcut connections benefits from an appropriate learning rate, which helps to avoid the issue of vanishing gradients.

The effect of the learning rate on each layer of the ResNet18 model is captured by the equation below:

(2) $$\theta_{t+1}=\theta_{t}-\eta \nabla L(\theta_{t}),$$

where $\theta_{t}$ represents the parameters of the current model, $\theta_{t+1}$ represents the parameters of the updated model, and *η* is the learning rate, which controls the step size of parameter updating. $\nabla L(\theta_{t})$ is the gradient of the loss function.

For example, the influence on the convolutional connected layers can be represented by Equations (3) and (4).

(3) $$W_{conv_{t}}^{\left(l\right)}=W_{conv_{t}}^{\left(l\right)}-\eta \nabla L_{conv_{t}}^{\left(l\right)}(W_{conv_{t}}^{\left(l\right)}),$$

(4) $${W^{\left(l\right)}}_{fc_{t+1}}={W^{\left(l\right)}}_{fc_{t}}-\eta \nabla {L^{\left(l\right)}}_{fc}\left({W^{\left(l\right)}}_{fc_{t}}\right).$$

In a comprehensive and rigorous attempt to authenticate the efficacy of the ResNet18 neural network model within the complex realm of image classification, a painstaking selection of five preeminent and canonical models is executed. These models include the Recurrent Neural Network (RNN) [47], the Convolutional Neural Network (CNN) [2], the Long Short-Term Memory network (LSTM) [48], the Generative Adversarial Network (GAN) [49], and the Visual Geometry Group network (VGG) [50]. Subsequently, the FASHION-MNIST dataset in synergy with the LIBWONN optimization algorithm is harnessed for the training process. The FASHION-MNIST dataset, characterized by its distinct features relevant to fashion-focused image classification tasks, provides an opportune medium for evaluating the models’ performance. In Table 1, the experimental results show that ResNet18 performed the best in both training and testing, with a test accuracy of 95.33%, which is about 4.28% higher than the worst-performing GAN. CNN and VGG showed performances close to ResNet18 but were slightly inferior. RNN and LSTM performed worse, with accuracies about 2% to 3% lower than ResNet18. GAN performed the worst, with higher training and testing losses. The residual connections in ResNet18 effectively avoid the vanishing gradient problem in deep networks, ensuring faster convergence and higher accuracy. Other models, such as RNN and LSTM, are more suitable for sequential data and perform worse in image classification tasks compared to CNN and ResNet. Although VGG also achieved good results, it lacks the residual structure of ResNet18, leading to a slightly worse training and testing performance. Therefore, ResNet18, with its unique structural advantages, outperforms other classic neural network models in handling image classification tasks.

### 2.2. Black Widow Optimization Algorithm

Inspired by the biological characteristics of the black widow spider, the black widow optimization (BWO) algorithm is a novel nature-inspired metaheuristic optimization method. The algorithm simulates the strategies male black widow spiders use to locate females, combining pheromone-guided actions with movement strategies within their web to effectively search and optimize solutions in complex problem spaces. The specific principles of the two strategies employed by the BWO are explained as follows:

1. Movement Strategy

The movement strategy of the black widow spider is one of its mobility strategies; it can be abstracted into linear and spiral movements within its web.

(5) $$\vec{x_{i}}\left(t+1\right)=\left\{\begin{array}{c} \vec{x}*\left(t\right)-m\vec{x_{r_{1}}}\left(t\right),\;if\;rand()\leq 0.3,\\ \vec{x}*\left(t\right)-\cos(2\pi \beta )\vec{x_{i}}(t),\;in\;other\;case \end{array}\right\},$$

where $\vec{x_{i}}\left(t+1\right)$ is the new position of the current search agent, $\vec{x}*\left(t\right)$ is the best search agent from the previous iteration, *m* denotes a randomly generated floating-point number within the range [0.4, 0.9], $\vec{x_{i}}(t)$ represents the *i*-th search agent, and *β* denotes a random floating-point number within the range [−1.0, 1.0].

When the spider moves linearly, it follows a determined direction for precise and effective searching in the current region. In contrast, spiral movement expands its search range, enhancing global exploration and helping to avoid local optima. The algorithm implements the black widow spider’s movement using a formula with random floating-point numbers, where m controls speed, and added randomness further enhances search diversity. By strategically combining both linear and spiral movements, the algorithm balances local exploitation and global exploration, ultimately improving its ability to solve complex optimization problems efficiently.

2. Pheromone

Pheromones play a crucial role in the mating process of spiders, as male spiders tend to prioritize female spiders with higher pheromone levels. The formula for calculating pheromones is as follows:

(6) $$pheromone\left(i\right)=\frac{fitness_{max}-fitness\left(i\right)}{fitness_{max}-fitness_{min}},$$

where $fitness_{max}$ and $fitness_{min}$ are the worst and best fitness values in the global iteration, respectively. $fitness\left(i\right)$ is the fitness of *i*-th search agent.

(7) $${\vec{x}}_{i}\left(t\right)=\vec{x}*\left(t\right)+\frac{1}{2}\left[{\vec{x}}_{r_{1}}\left(t\right)-{\left(-1\right)}^{\sigma }*{\vec{x}}_{r_{2}}\left(t\right)\right],$$

When the pheromone level is less than or equal to 0.3, the search agent $\vec{x_{i}}(t)$ is updated by Equation (3). ${\vec{x}}_{r_{1}}\left(t\right)$ and ${\vec{x}}_{r_{2}}\left(t\right)$ are the random integers generated within the range from 1 to the maximum size of the search agents, $\sigma \in \left\{0,1\right\}$. Additionally, the pseudocode for the BWO algorithm can be found in Algorithm 1.
**Algorithm 1: BWO****Input: MaxIter, pop, dim**
  **Operation**

/* Initialization */1.**Initialize:** MaxIter, pop, dim
2.**Initialize:** parameters *m* and β
/* Training Starts */3.**while** iteration < Max Number of Iterations do
4.     **if** random < 0.3 **then**5. 6. 7. 8. 9. 10. 11. 12. 13. 14. 15.            $X_{i}^{n\mathrm{ew}}\leftarrow X^{*}(t)-mX_{r1}(t)$    **else**             $X_{i}^{new}\leftarrow X^{*}(t)-cos(2\pi \beta )x_{i}(t)$    **end if**     Calculate the pheromone for each search agent using the specified Equation (6)    Revise search agents with low pheromone values using its Equation (7)    Calculate $X^{new}$ fitness value of the new search agents    **if**
$X^{new}<X^{*}$
**then**             $X^{*}\leftarrow X^{new}$ **end if**    iteration←iteration+1
16.**end while**/* Operation Ending */**Output:** $X^{*}$, the best optimal solution

The algorithm takes as input the maximum number of iterations (MaxIter), population size (pop), and dimension (dim). It first initializes the parameters *m* and *β*. During the iterative process, the algorithm selects different operations based on a set probability to maintain population diversity and search capability.

In each iteration, the pheromone of each individual is calculated, which reflects the individual’s fitness. Individuals with lower pheromone values update their positions to avoid becoming trapped in the local optima. After updating, the fitness values are recalculated, and the global best solution is updated if a better solution is found. After the iterations end, the algorithm outputs the best solution X*. This process simulates the hunting behavior of black widow spiders to efficiently search for the global optimum.

### 2.3. Lagrange Interpolation Method

The BWO algorithm has excellent performance in solving complex optimization problems, but it still has some limitations, as it is sensitive to critical parameters such as the population size during initialization, the maximum number of iterations, and the dimensionality of the problem. In some cases, the BWO algorithm may encounter local optima and exhibit an unstable convergence speed, which can lead to a decrease in solution quality and a slowdown in optimization speed.

To address the issues in optimization algorithms, F. Miao, Y. Wu et al. propose a quadratic interpolation whale optimization algorithm for solving high-dimensional feature selection problems [26,27], while Z. Li, S. Li et al. propose a novel cubic interpolated beetle antennae search (CIBAS)-based robot arm calibration algorithm [51].

Therefore, this study also introduces mathematical interpolation methods to optimize the BWO algorithm. We select six interpolation methods (Lagrange interpolation, Newton interpolation [42], spline interpolation [43], quadratic interpolation [44], linear interpolation [45], and Chebyshev interpolation [46]) and conduct extensive comparative experiments on the FASHION-MNIST dataset with the ResNet18 model. From the data in Table 2, it can be clearly seen that Lagrange interpolation performs the best in optimizing the black widow optimization (BWO) algorithm. It achieves the lowest training and testing losses, as well as the highest training and testing accuracies, indicating superior convergence and generalization abilities. Furthermore, this method achieves 0.95 in precision, recall, and F1 score, resulting in the best overall classification performance. In contrast, the other interpolation methods show slightly lower testing accuracy and F1 scores, with quadratic and linear interpolation performing particularly poorly. This indicates that Lagrange interpolation is significantly more effective in optimizing the BWO algorithm, leading to faster convergence, stronger generalization, and more stable classification performance across various problem domains, especially in complex and large-scale optimization tasks.

Lagrange interpolation is employed to enhance the position-updating process of each spider, thereby further optimizing the BWO algorithm. When a spider needs to update its position toward the global optimum, a new position is calculated through Lagrange interpolation, which adopts several known optimal spider positions. Then, the new position is compared with the global optimal position to decide whether to update. This approach mitigates excessive jumps in the spider’s movement, enhancing the algorithm’s convergence and robustness, which helps avoid local optima and improve global search performance.

The Lagrange interpolation polynomial is given by the following:

(8) $$L\left(x\right)=\sum_{j=0}^{k}y_{i}l_{j}\left(x\right).$$

Each *l_j_* (*x*) is a Lagrangian polynomial, which is expressed as follows:

(9) $$l_{j}\left(x\right)=\prod_{i=0,i\neq j}^{k}\frac{x-x_{i}}{x_{j}-x_{i}}=\frac{x-x_{0}}{x_{j}-x_{0}}\ldots \frac{x-x_{j-1}}{x_{j}-x_{j-1}}\frac{x-x_{j+1}}{x_{j}-x_{j+1}}\ldots \frac{x-x_{k}}{x_{j}-x_{k}},$$

where *x_j_* is the position of the independent variable and *y_j_* is the value of the function at this position.

The Lagrange interpolation method calculates the value of an unknown point by using the values of three known points. The steps are roughly as follows:

1. Determining the coordinate information of the three known points.

(10) $$A=\left(x_{0},y_{0}\right),B=\left(x_{1},y_{1}\right),C=\left(x_{2},y_{2}\right),$$

2. Calculating the Lagrange basis function.

(11) $$l_{0}\left(x\right)=\frac{\left(x-x_{1}\right)\left(x-x_{2}\right)}{\left(x_{0}-x_{1}\right)\left(x_{0}-x_{2}\right)},$$

(12) $$l_{1}\left(x\right)=\frac{\left(x-x_{0}\right)\left(x-x_{2}\right)}{\left(x_{1}-x_{0}\right)\left(x_{1}-x_{2}\right)},$$

(13) $$l_{2}\left(x\right)=\frac{\left(x-x_{0}\right)\left(x-x_{1}\right)}{\left(x_{2}-x_{0}\right)\left(x_{2}-x_{1}\right)}.$$

3. The Lagrange interpolation polynomial is obtained by adding three equations together.

(14) $$L\left(x\right)=y_{0}l_{0}\left(x\right)+y_{1}l_{1}\left(x\right)+y_{2}l_{2}\left(x\right).$$

By using the Lagrange interpolation method to optimize the BWO algorithm (LIBWONN), its convergence speed and local search capability are enhanced. Using Lagrange interpolation, a new optimal fitness is derived from the current optimal fitness, global optimal fitness, and previous optimal fitness. Furthermore, the new optimal fitness is evaluated against the global optimal fitness to determine if an update to the global optimal fitness is necessary.

### 2.4. The Model Design Based on the LIBWONN Method

**Figure 4:**
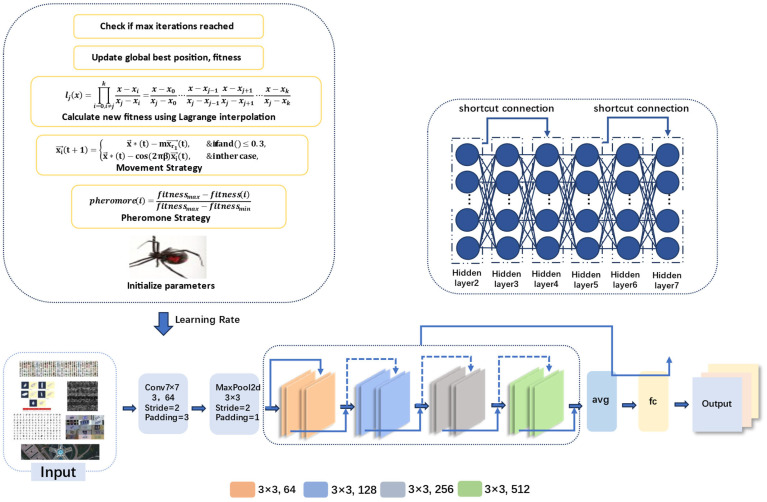
The model structure of the LIBWONN method.

We employ the LIBWONN algorithm to dynamically adjust the learning rate of the ResNet18 model, which improves training efficiency. This approach allows the model to converge quickly in the early stages and gradually reduce the learning rate as it approaches the optimal solution, thereby reducing training time and the number of iterations. Additionally, this approach helps avoid local optima and enhances the model’s overall performance. The adaptive learning rate also improves model stability, reducing the risk of overfitting and enhancing generalization ability. Meanwhile, dynamic adjustment simplifies hyper-parameter tuning, reduces manual intervention, and increases training efficiency. Moreover, the model structure is as follows:

The model optimization process shown in Figure 4 is based on the black widow optimization algorithm (LIBWONN), which simulates the hunting behavior of black widow spiders in nature to search for the optimal learning rate. The training data comes from a public dataset, which is normalized before being used to train a deep neural network. After each training session, the validation loss is calculated using a validation set. This loss value acts as a pheromone in the model and serves as a metric to evaluate the quality of the current learning rate, guiding the individuals during the optimization process.

The core of the black widow optimization algorithm lies in simulating the predatory behavior of a spider population for search and optimization. Each individual (i.e., spider) represents a potential learning rate, randomly selected within a defined boundary range. After training the model, the validation loss corresponding to each individual is calculated and used as its fitness value. The higher the fitness, the stronger the pheromone released, attracting other individuals to move closer and accelerating convergence toward the optimal solution.

In each iteration, individuals update their positions not only under the guidance of the current best and global best solutions, but also by incorporating Lagrange interpolation to enhance the algorithm’s search capability and convergence performance. Unlike traditional update strategies that rely solely on current fitness values for local adjustments, the Lagrange interpolation mechanism leverages trend information derived from historical optimal solutions to predict potentially better learning rate positions. Specifically, during the optimization process, three key points are recorded: the best learning rate and its corresponding validation loss from the previous iteration, the best learning rate and its loss from the current iteration, and the global best learning rate with its loss. These three points are treated as inputs to the Lagrange interpolation function, which constructs a polynomial to estimate the functional relationship between the learning rate and loss.

The interpolated result yields a predicted learning rate position that is likely to produce a lower validation loss. This position is then evaluated through training, and if its corresponding loss outperforms the current global best, it is adopted as the new global optimum and releases stronger pheromones to guide future searches. Since the interpolation integrates multiple historically optimal points and captures the underlying optimization trend, it not only improves search accuracy and speeds up convergence but also enhances the algorithm’s ability to escape local optima and maintain population diversity. As a result, the LIBWONN algorithm demonstrates superior performance in dynamically tuning the learning rate, ultimately improving model generalization and classification accuracy.

In this way, during each iteration, the LIBWONN algorithm not only relies on traditional fitness evaluation and position updates but also uses Lagrange interpolation to predict potentially better solutions. This accelerates the search for the optimal learning rate and helps the neural network achieve better performance during training. Ultimately, the optimized model performs significantly better on the validation set, effectively improving classification accuracy and generalization ability. Moreover, by incorporating historical optimal points into the interpolation process, the algorithm enhances its ability to escape local optima and explores a wider solution space more effectively.

Moreover, the pseudocode of the LIBWONN algorithm is in Algorithm 2.
**Algorithm 2: LIBWONN****Input:** MaxIter, pop, dim
**Operation**
/* Initialization */1.**Initialize:** MaxIter, pop, dim
2.**Initialize:** parameters *m* and β
/* Training Starts */3.**while** iteration < Max Number of Iterations do
4.     **if** random < 0.3 **then**5. 6. 7. 8. 9. 10. 11. 12. 13. 14. 15. 16. 17. 18. 19.            $X_{i}^{new}\leftarrow X^{*}(t)-mX_{r1}(t)$    **else**            $X_{i}^{new}\leftarrow X^{*}(t)-cos(2\pi \beta )x_{i}(t)$    **end if**         Calculate the pheromone for each search agent using the specified Equation (6)    Revise search agents with low pheromone values using its Equation (7)    Calculate $X^{new}$ fitness value of the new search agents    **if**
$X^{new}<X^{*}$
**then**             $X^{*}\leftarrow X^{new}$    **end if**     $f_{new}\leftarrow Lagrange\_Interpolation(f_{old},f_{best},f_{current})$     **if**
$f_{new}<f_{best}$
**then**             $f_{best}\leftarrow f_{new}$     **end if**     iteration←iteration+1
20.**end while**/* Operation Ending */**Output:** $X^{*}$, the best optimal solution

The primary steps of the enhanced LIBWONN algorithm are as follows:

Step 1: Initialize the population parameters, such as population size, maximum iterations, and population boundary range, randomly initializing the optimal position and optimal fitness. We calculate the pheromone value by using Equation (2) based on the pheromone strategy.

Step 2: Update spider positions by using Equation (1) according to the movement strategy. If the pheromone value is less than 0.3, the pheromone strategy updates the current individual position by using Equation (2).

Step 3: Adjust the current individual’s position and fitness.

Step 4: Update the global optimal position, optimal fitness, and pheromone.

Step 5: Use the current optimal fitness, global optimal fitness, and previous optimal fitness to calculate a new fitness value via Lagrange interpolation. Evaluate it against the global optimal fitness and update the global optimal fitness if needed.

Step 6: Verify whether the maximum iterations are completed. If not, proceed back to Step 2; otherwise, halt the iteration and return the optimal position and fitness.

## 3. Experiments and Analyses

### 3.1. Dataset

A total of six public datasets are used in the experiments, which cover various scenarios such as clothing, handwritten digits, and street-view images. Their details are as follows:

1. FASHION-MNIST [52]: A clothing image dataset with grayscale images sized 28 × 28 pixels contains 10 different clothing categories, such as T-shirts, trousers, dresses. The training set contains 60,000 samples, while the test set comprises 10,000 samples.
2. MNIST [53]: This dataset for handwritten digit recognition contains 28 × 28 pixel images of digits from 0 to 9, with 60,000 samples for training and 10,000 samples for testing.
3. Intel Image Classification [54]: This dataset includes images from six different categories, like buildings, forests, glaciers, mountains, oceans, and cities. All images are standardized to 150 × 150 pixels. The training set contains 14,034 samples, and the testing set has 3,000 samples.
4. SVHN [55]: A digit recognition dataset has street-view images with 32 × 32 pixels. It includes 10 categories corresponding to the digits 0–9, and has 73,254 training images and 26,032 test images.
5. RICE [56]: It covers five common rice varieties: Arborio, Basmati, Ipsala, Jasmine, and Karacadag. Image dimensions are 224 × 224 pixels with a total of 3,800 images.
6. CIFAR10 [57]: This dataset includes images from 10 different categories: Airplane, automobile, bird, cat, deer, dog, frog, horse, ship, and truck. The images are 32 × 32 pixels with a training set of 50,000 images and a test set of 10,000 images.

### 3.2. Base Models

To validate the performance of the LIBWONN algorithm, nine novel and advanced optimization algorithms are selected for comparative experiments.

1. DOA: Dream Optimization Algorithm (DOA) is a novel metaheuristic algorithm inspired by human dreams. The algorithm combines a basic memory strategy with a forgetting and replenishing strategy to balance exploration and exploitation.
2. ALA: Artificial Lemming Algorithm (ALA) is a biologically inspired metaheuristic algorithm inspired by the four basic behaviors of lemmings in nature: long migrations, digging holes, foraging for food, and avoiding predators. The algorithm simulates the survival strategies of lemmings in complex environments, providing an effective search method for solving optimization problems.
3. SDO: Sled Dog Optimizer (SDO) is mainly inspired by the various behavior patterns of sled dogs, focusing on simulating the processes of dogs pulling sleds, training, and retiring to construct a mathematical model.
4. CBSO: Connected Banking System Optimizer (CBSO) is a population-based optimization algorithm that belongs to a multi-stage search strategy. It is inspired by the interconnectedness of banking systems, where different banks are connected in various ways, facilitating transactions and submissions.
5. PSEQADE: Quantum Adaptive Population State Evaluation Differential Evolution Algorithm (PSEQADE) is an improved quantum heuristic differential evolution algorithm. It adopts a quantum adaptive mutation strategy to reduce excessive mutation and introduces a population state evaluation framework to enhance convergence accuracy and stability.
6. COVIDOA: Coronavirus Optimization Algorithm (COVIDOA) is an evolutionary algorithm that simulates the biological lifecycle. It is inspired by the behavior of organisms at different stages such as growth, reproduction, and adaptation. The algorithm simulates the evolutionary process of individuals from youth to adulthood, adapting through mutation, recombination, selection, and reproduction based on environmental changes, thereby balancing global and local search capabilities.
7. SASS: Social-Aware Salp Swarm Algorithm (SASS) is a population-based optimization algorithm inspired by the behavior of sand particles in a sandstorm. It mainly simulates the collective movement of sand particles under the influence of wind to perform global optimization. The goal of SASS is to improve collaboration among individuals in the group using a social awareness model, enhancing the balance between exploration and exploitation in the search process.
8. LSHADE-cnEpSin: Latent Search Strategy Adaptive Differential Evolution with Compound Neighborhood-based Epistemic Population for Sine Function (LSHADE-cnEpSin) is an improved differential evolution algorithm. It enhances optimization performance, especially for high-dimensional complex problems, by using an adaptive mutation strategy and a control mechanism that balances global and local search.
9. AGSK: Adaptive Gaining Sharing Knowledge (AGSK) is an algorithm that simulates the human knowledge-sharing process. It enhances global search capability and local search efficiency by introducing an adaptation strategy based on successful historical positional information, making it suitable for solving complex optimization problems.
10. BOA: The Bobcat Optimization Algorithm (BOA) is a bio-inspired metaheuristic algorithm that simulates the natural hunting behavior of bobcats. It enhances the balance between global exploration and local exploitation by modeling two phases: the bobcat’s movement towards its prey (exploration) and the chase process to catch its prey (exploitation). BOA’s dual-phase position update strategy improves convergence speed and solution quality, making it effective for solving high-dimensional, complex, and constrained optimization problems.
11. WOA: The Wombat Optimization Algorithm (WOA) is a bio-inspired metaheuristic algorithm that simulates the foraging behavior of wild wombats and their evasive maneuvers against predators. The algorithm models two phases: the wombat’s position changes during foraging (exploration) and its movements when diving into tunnels to escape predators (exploitation), effectively balancing global search and local search.

### 3.3. Performance Verification

In an effort to rigorously validate the efficacy of the LIBWONN algorithm in adaptively optimizing the learning rate of the ResNet18 model, a set of comparative experiments are meticulously carried out. In these experiments, the LIBWONN algorithm is contrasted with nine other cutting-edge optimization algorithms. The algorithms selected are thus emblematic of the deep-learning domain, ensuring a comprehensive and probative evaluation of the LIBWONN algorithm’s performance in the optimization of RenNet18 model. The experiments involve six public datasets to ensure the comprehensiveness and reliability of the evaluation results. The chosen datasets cover various fields and tasks, which allows for testing the algorithms’ performance through diverse applications and validating the applicability of the LIBWONN algorithm.

**Figure 5:**
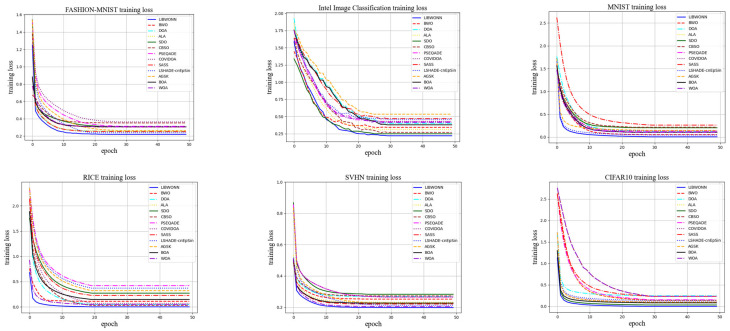
The training loss images on six datasets.

In Figure 5, the LIBWONN algorithm performs excellently on multiple datasets. Specifically, LIBWONN shows the fastest convergence speed and achieves convergence by the 10th iteration on the FASHION-MNIST, MNIST, and SVHN datasets, where the final loss value reaches its minimum. This indicates that the LIBWONN algorithm is able to effectively capture the features of the data on these relatively simple datasets.

On the Intel Image Classification dataset, compared with the WOA model, LIBWONN’s initial convergence speed and final loss value are slightly inferior by 1.08%. This is mainly attributed to the higher complexity and diversity of the dataset, which requires more iterations for the model to adequately capture the data patterns. Despite its relatively weaker performance on this dataset, LIBWONN still demonstrates a good convergence capability and maintains a relatively low final loss value overall.

Overall, the LIBWONN model performs exceptionally well across multiple datasets, especially in tasks involving FASHION-MNIST, MNIST, and SVHN, where its accuracy reaches above 98%. Its efficient convergence speed and low loss values highlight its effectiveness in image classification. Moreover, when dealing with more complex datasets, LIBWONN also shows strong adaptability, indicating promising potential for further optimization and exploration in future research.

**Figure 6:**
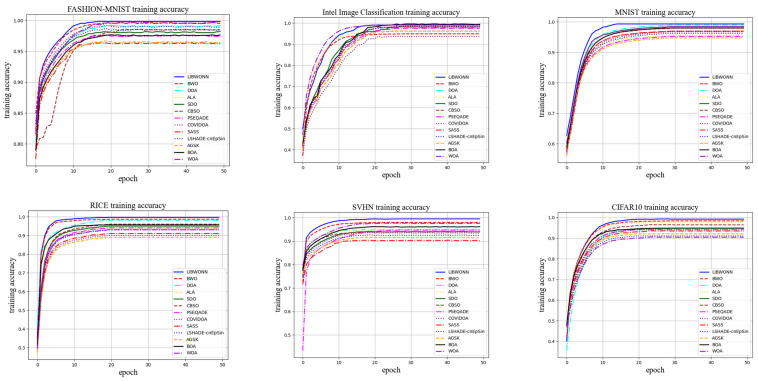
The training accuracy images on six datasets.

In Figure 6, we present a comparison of the training accuracy between different optimization algorithms and the proposed LIBWONN algorithm across five datasets. The results clearly demonstrate that LIBWONN performs exceptionally well, achieving high accuracy, requiring fewer training iterations, and exhibiting rapid convergence. Additionally, compared to the other optimization algorithms, LIBWONN offers enhanced stability and smoother training curves.

Notably, on the MNIST and SVHN datasets, the LIBWONN algorithm attains high accuracy within a short training time, highlighting its capability for fast convergence. However, on the Intel Image Classification dataset, its training accuracy exhibits more noticeable fluctuations. This can be attributed to the dataset’s greater diversity and complexity, as it comprises six different scene categories and intricate image structures, making generalization more challenging for the optimization algorithm. Factors such as noise, imbalanced sample distribution, and the limited size of the training dataset may lead the model to learn incorrect patterns, thereby causing accuracy fluctuations. In convolutional neural networks trained for image classification, dataset imbalance can further contribute to such variations. Nevertheless, when using models like ResNet, accuracy fluctuations typically smooth out over time rather than displaying random oscillations, which aligns with our expected trend.

Overall, the LIBWONN algorithm demonstrates outstanding performance across multiple datasets, particularly in terms of rapid convergence. However, when applied to complex datasets such as Intel Image Classification, the impact of dataset diversity and complexity on the model’s generalization ability must be carefully considered. By fine-tuning optimization parameters, employing data augmentation techniques, and leveraging other strategies, the model’s stability and accuracy can be further enhanced.

(15) $$accuracy=\frac{TP+TN}{TP+TN+FP+FN},$$

(16) $$precision=\frac{TP}{TP+FP},$$

(17) $$recall=\frac{TP}{TP+FN},$$

(18) $$F1-score=\frac{2\times precision\times recall}{precision+recall},$$

(19) $$W=\min\left(\sum_{D_{i}>0}R_{i},\sum_{D_{i}<0}R_{i}\right),$$

(20) $$Q=\frac{12n}{k(k+1)}\sum_{j=1}^{k}{\bar{R}}_{j}^{2}-3n(k+1),$$

*TP* (true positive): the prediction matches the actual positive class. *TN* (true negative): the prediction matches the actual negative class. *FP* (false positive): the prediction is positive, but the actual class is negative. *FN* (false negative): the prediction is negative, but the actual class is positive.

Accuracy, the most intuitive metric, represents the proportion of correct predictions out of all the samples. However, it does not always provide a complete picture of a model’s performance, particularly in imbalanced datasets where errors in the minority class have a minimal impact on overall accuracy. To quantitatively verify the performance of the LIBWONN algorithm, we select several evaluation metrics such as accuracy, precision, recall, and *F*1-*score*. These metrics are standard in the field of machine learning and are used to comprehensively assess the model’s predictive capabilities.

Precision measures the ratio of true positive predictions to the total positive predictions made by the model. This metric shows how well the model identifies actual positive cases. A high precision score means the model is more careful in predicting positives, reducing the likelihood of false positives.

Recall evaluates the percentage of true positives among all actual positive samples, reflecting the model’s ability to detect positive instances. A high recall means the model is adept at identifying all positive samples, thereby decreasing false negative errors.

*F*1-*Score*, being the harmonic mean of the precision and recall, is used to assess the balance between these metrics. It reaches its highest value of 1 when the precision and recall are equal, otherwise it diminishes. This metric is crucial in applications where both precision and recall need to be taken into account.

*p*-value represents the probability of observing the current data or more extreme results under the assumption that the null hypothesis is true, serving as a measure of the evidence against the null hypothesis. A lower *p*-value indicates a lower likelihood of the null hypothesis being true, providing a basis for its rejection. Typically, if the *p*-value is less than the significance level (e.g., 0.05), the result is considered statistically significant. This allows researchers to make data-driven decisions and draw conclusions about the relationships between variables. The mathematical formulas for the Wilcoxon signed-rank test and the Friedman test are shown in Equations (19) and (20), respectively. After standardization, hypothesis testing is conducted to validate the assumptions and ensure reliable results.

By thoroughly evaluating these metrics, we can better understand the performance of the LIBWONN algorithm across various datasets, as well as its potential and limitations in real-world applications, providing valuable insights into its practical applicability and robustness in different scenarios.

Table 2, Table 3, Table 4, Table 5, Table 6, Table 7 and Table 8 show the performance metrics of each benchmark model on six public datasets. In terms of the three comprehensive performance indicators, namely precision, recall, and F1-score, LIBWONN achieves the best results, which indicates that the LIBWONN algorithm has strong learning capabilities and significant advantages in classification accuracy, recall, and overall performance. This demonstrates that LIBWONN is able to effectively capture patterns and make accurate predictions across various datasets. Its superior performance in both precision and recall highlights its ability to balance the trade-off between minimizing false positives and false negatives, making it a highly reliable model for real-world applications.

In the signed-rank test, the *p*-value for most cases is less than 0.05, indicating that there is a significant difference between the other algorithms and LIBWONN. This suggests that the superior performance of LIBWONN is not due to random chance but is statistically significant. A *p*-value smaller than 0.05 typically means that the null hypothesis can be rejected, confirming that LIBWONN consistently outperforms the other algorithms in terms of accuracy, recall, and F1-score. This strengthens the validity of LIBWONN as a more effective and reliable algorithm for solving optimization and classification tasks.

LIBWONN performs exceptionally well on the MNIST and RICE datasets, with its training and testing loss being relatively low, and its accuracy exceeding 99%. All metrics reach their highest levels, showing a 3.44% improvement in performance on the training set compared to BWO, followed by a 2.16% improvement on the test set. Additionally, LIBWONN achieves excellent results on relatively complex datasets such as SVHN, Fashion-MNIST, and Intel Image Classification. Although its performance slightly declines on the texture-rich Fashion-MNIST and the diverse natural scenes of the Intel Image Classification dataset, LIBWONN still maintains a low loss, high accuracy, and outstanding precision, recall, and F1 scores. These results demonstrate LIBWONN’s consistent performance across a wide range of datasets. In the SVHN dataset’s training set, LIBWONN trails BWO by just 0.37%.

### 3.4. Testing Functions

The CEC 2017 and CEC 2022 testing functions, presented at the IEEE Congress on Evolutionary Computation, serve as essential benchmarks for evaluating optimization algorithms. The CEC 2017 functions encompass various optimization problems, such as unimodal, multimodal, composite, and dynamic types. These functions are designed to test the algorithm’s performance under different complexities and characteristics, thus evaluating its capabilities in both static and dynamic environments. The CEC 2022 testing function introduces complex challenges, which includes intricate multimodal function, high-dimensional function, irregular function, discontinuous function, and constrained optimization problems. These new features are intended to better reflect the challenges of modern applications, which test algorithms’ performance in high-dimensional spaces and complex landscapes, as well as under constraints. CEC 2022 emphasizes improving the robustness and adaptability of algorithms, thus providing a more comprehensive testing environment.

- Unimodal functions: Characterized by a single global optimum, they are used to test an algorithm’s local search proficiency and convergence speed. They are effective in evaluating the algorithm’s efficiency and accuracy in basic situations.
- Multimodal functions: Characterized by several local optima, they test the performance of an algorithm to navigate out of local optima as well as its global search capability. These functions are adopted to measure the performance of the algorithm in complex and nonlinear environments.
- Composite functions: They are composed of multiple functions with different characteristics, which simulate the complexity and diversity of real-world problems. They are adopted to verify the algorithm’s adaptability when handling mixed features and multiple levels of difficulty.
- Dynamic change functions: Their objective function changes over time, and are used to evaluate the algorithm’s tracking and adaptability in dynamic environments. They are especially suitable for testing an algorithm’s ability to maintain its performance under continuously changing conditions.

The characteristics of these test functions lie in their diversity and complexity, thus allowing researchers to test the performance of algorithms through different dimensions and scenarios. The design of these functions takes into account various factors, including smoothness, differentiability, and the number of local optima. These factors directly affect the performance of optimization algorithms during the solution process. Moreover, Table 9 and Table 10 summarize the basic information regarding the CEC 2017 and CEC 2022 testing functions in our paper, including function numbers, function names, and global minimum values.

**Figure 7:**
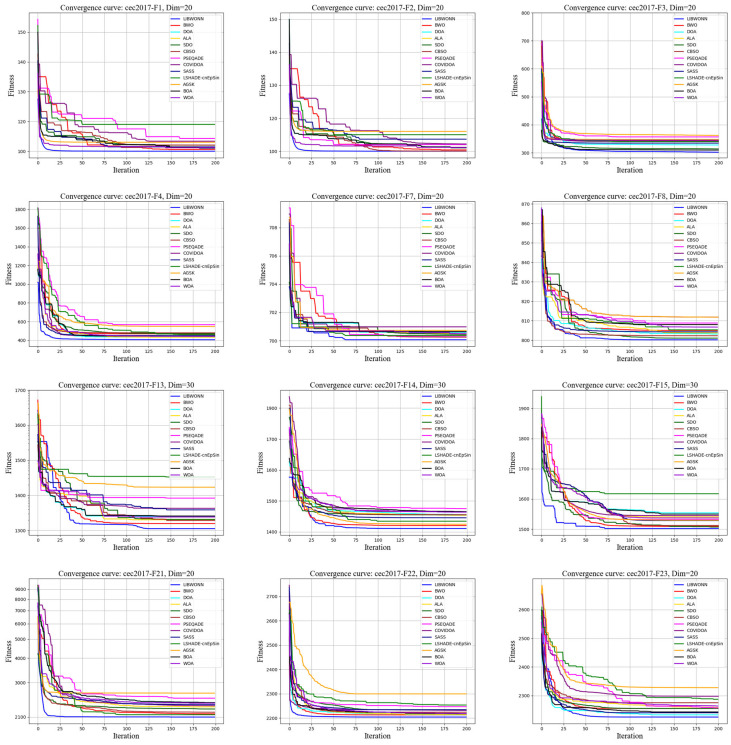
CEC2017 testing functions.

**Figure 8:**
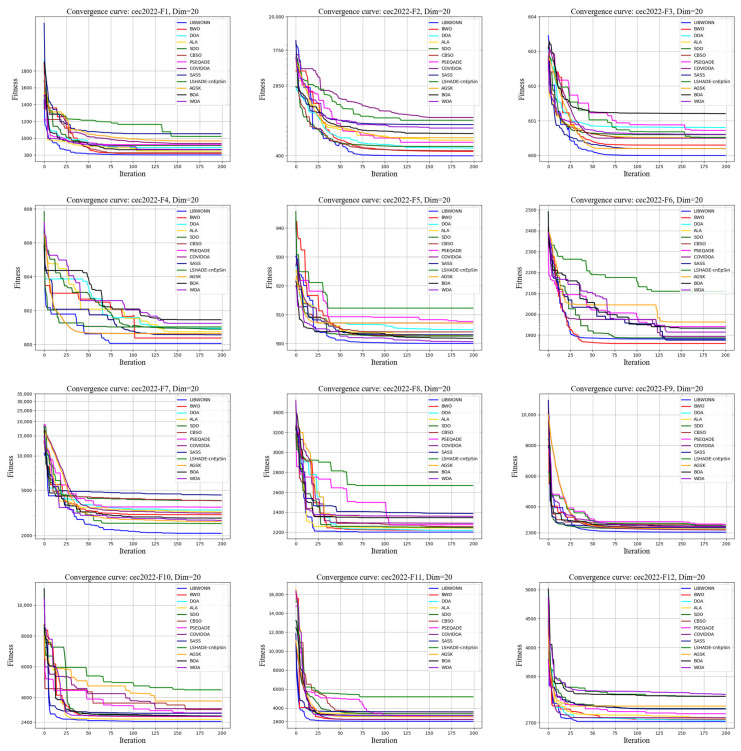
CEC2022 testing functions.

To thoroughly evaluate the performance of different optimization algorithms on the CEC test functions, we conducted multiple experiments using various algorithms to iteratively solve several test functions and plotted their fitness value curves. Figure 7 and Figure 8 illustrate the fitness changes over 200 iterations for different algorithms on the CEC2017 and CEC2022 test functions, providing a clear basis for subsequent analysis.

Figure 7 shows that LIBWONN and BWO exhibit rapid decreases in their fitness values within the first 50 iterations, demonstrating fast convergence speeds and strong search capabilities. Additionally, LIBWONN’s fitness curve shows smaller fluctuations, indicating better stability. This fast convergence makes these algorithms especially suitable for time-sensitive optimization problems, as they can quickly locate regions near the optimal solution. Although LIBWONN performs slightly worse than SDO and CBSO on the F3 and F7 test functions, it remains highly competitive overall and excels in handling complex and large-scale functions. Its efficient and stable characteristics give it advantages in practical applications, showing good robustness against parameter variations and environmental disturbances.

Figure 8 presents the performance of LIBWONN on the CEC2022 test functions, where it consistently demonstrates a rapid fitness decline, reflecting its excellent convergence ability and stability. The algorithm effectively balances exploration and exploitation, avoiding local optima and ensuring global optimization. Compared with the other algorithms, LIBWONN outperforms them in both convergence speed and final fitness values, showing strong adaptability, especially suited for high-dimensional and complex optimization tasks. Moreover, LIBWONN maintains stable performance across different test environments, highlighting its potential for applications in dynamic and complex scenarios.

To further quantify the algorithm’s performance, we evaluated it using three metrics: average value, best value, and standard deviation. The average fitness value reflects overall stability and reliability across multiple runs; a lower average indicates a consistent good performance. The best value measures the algorithm’s ability to find the optimal solution, while the standard deviation assesses the variability of its results, with smaller values indicating greater stability. Together, these metrics show that LIBWONN performs excellently across the various test functions, confirming its efficiency and broad applicability in complex optimization problems.

Combining the fitness curves and quantitative metrics, the LIBWONN algorithm demonstrates significant advantages in fast convergence, superior stability, and strong adaptability across multiple test functions, proving its potential and value in solving real-world complex optimization challenges.

The best value refers to the lowest fitness value achieved by the algorithm on multiple iterations, the optimal solution the algorithm can reach in a given environment. Moreover, the best value of the LIBWONN algorithm directly reflects its efficiency in exploring the solution space. A lower best value indicates that the algorithm is capable of effectively finding solutions close to the global optimum.

The standard deviation is an indicator for assessing the variability of the algorithm’s results, thus reflecting the stability of LIBWONN on multiple runs. A smaller standard deviation shows a higher consistency of the algorithm’s results in different experiments, which indicates that LIBWONN can maintain a stable optimization performance in the face of various problems. It is particularly important in practical applications to ensure the reliability and reproducibility of the algorithm.

From an extensive analysis of these three metrics, we can thoroughly evaluate the performance of the LIBWONN optimization algorithm and affirm its effectiveness in handling complex optimization tasks. These quantitative results provide us with a more solid theoretical foundation, thus aiding us in understanding the significant advantages of the LIBWONN algorithm in practical applications.

1. Average value (Ave):

(21) $$Ave=\frac{\sum_{i=1}^{n}x_{i}}{n},$$

where *x_i_* is the total number of samples.

2. Best value (Best):

(22) $$Best=\min\left(x_{1},x_{2},\ldots ,x_{n}\right).$$

3. Standard deviation (Std):

(23) $$Std=\sqrt{\frac{\sum_{i=1}^{n}{\left(x_{i}-\bar{x}\right)}^{2}}{n-1}}.$$

Each test function is uniformly set at 20 dimensions, except for F13, F14, and F15 in CEC2017, which are set at 30 dimensions.

The detailed results of the CEC2017 test functions are presented in Table 11. From the average values of each test function, it is evident that LIBWONN and BWO demonstrate superior performance across most functions, particularly for functions F1, F3, and F4. Their average values are consistently lower than those of the other algorithms, indicating that these two algorithms excel in optimizing these specific problems. This performance highlights LIBWONN and BWO’s strong ability to explore the solution space, suggesting that their algorithmic designs are highly effective in quickly locating regions near the optimal solution.

In terms of stability, the analysis of the standard deviation shows that LIBWONN and BWO generally exhibit low standard deviations, underscoring their high stability throughout the optimization process. For example, the standard deviations of LIBWONN and BWO remain within an acceptable range for the functions F1, F3, and F4, demonstrating consistent and reliable results.

The comparison of the best values further underscores the advantage of LIBWONN. In several of the test functions, LIBWONN achieves the best values, securing the first rank, which emphasizes its exceptional overall performance. This result confirms the efficacy of LIBWONN in optimization tasks and demonstrates its excellent adaptability and competitiveness across various complex problems.

Table 12 presents the detailed results of the CEC2022 test functions. Analyzing these results reveals that the LIBWONN algorithm continues to excel in several of the test functions, particularly F1 and F2, where its average fitness is notably lower than that of the other algorithms, further showcasing its optimization capabilities for complex problems. From the perspective of standard deviation, LIBWONN maintains relatively low standard deviation values, indicating remarkable stability. This suggests that LIBWONN can consistently deliver reliable optimization results across multiple experiments. In summary, LIBWONN’s performance in the CEC2022 test functions reinforces the advantages observed in CEC2017, confirming its continued effectiveness and reliability in solving complex optimization challenges.

### 3.5. Ablation Study and Sensitivity Analysis

In an attempt to validate the contribution of each component within the LIBWONN algorithm to the performance of the ResNet18 model, and thereby to illustrate the efficacy of LIBWONN, two ablation experiments are carried out during the training of the ResNet18 model on the FASHION-MNIST dataset: (1) LIBWONN is substituted with a conventional optimization approach employing a fixed learning rate, and (2) the Lagrange interpolation in LIBWONN is removed, and only the BWO algorithm is used for the optimization effectiveness of LIBWONN.

The experimental results indicate that LIBWONN exhibits significant performance advantages over the other optimization methods, as shown in Table 13. When replacing LIBWONN with a conventional optimization method using a fixed learning rate, both training and testing losses increased substantially, suggesting that a fixed learning rate fails to adapt effectively to the characteristics of the dataset, resulting in suboptimal model training. When removing the Lagrange interpolation and using only BWO for optimization, the testing accuracy improved to 92.50%, surpassing that of the fixed learning rate approach but still falling short of the 95.33% achieved by LIBWONN. This result demonstrates that while BWO effectively optimizes the learning rate, the absence of Lagrange interpolation reduces its precision and global search capability, preventing the model from achieving optimal performance.

**Figure 9:**
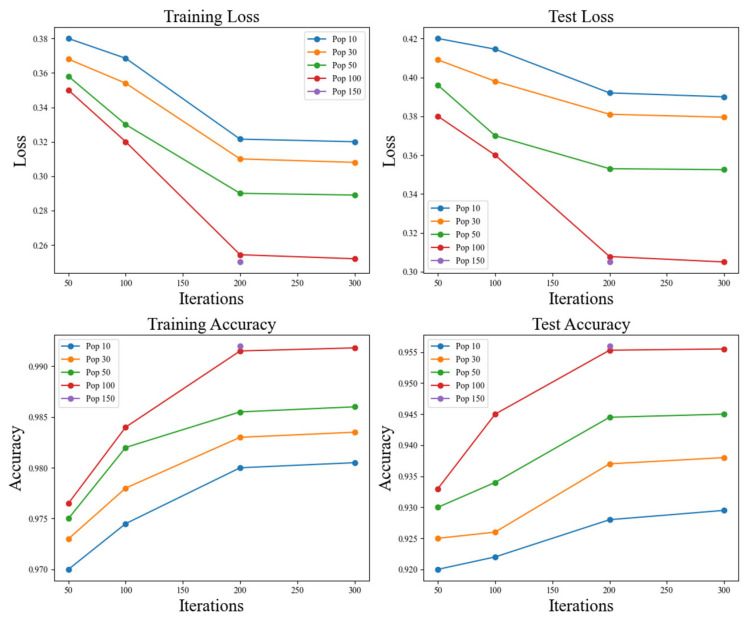
Line chart of sensitivity analysis results.

A sensitivity analysis experiment primarily checks the impact of population size and the number of iterations on the performance of LIBWONN in optimizing the ResNet18 model. The experimental results indicate that as the population size and the number of iterations increase, both the training loss and test loss gradually decrease, while the training accuracy and test accuracy continuously improve. This validates the optimization process. The result is shown in Table 14 and the line graph in Figure 9.

Specifically, when the population size reaches 100 and the number of iterations is set to 200, the training loss decreases to 0.2543, the test loss decreases to 0.3078, the training accuracy reaches 99.15%, and the test accuracy reaches 95.53%. In contrast, smaller population sizes (e.g., 10 or 30) and fewer iterations (e.g., 50 or 100) lead to a higher loss and lower accuracy, suggesting that a smaller search space and a shorter optimization process fail to sufficiently explore the optimal solution of the model.

However, when the population size and the number of iterations increase to 150 and 300, respectively, the reduction in training and test loss tends to level off, and the improvement in test accuracy becomes less significant. For example, with 200 iterations, increasing the population size from 100 to 150 results in only a slight test accuracy improvement from 95.53% to 95.60%. Similarly, with 300 iterations, increasing the population size from 100 to 150 raises the test accuracy only marginally from 95.55% to 95.62%. This indicates that once the population size and the number of iterations reach a certain threshold, further increasing computational resources provides limited optimization benefits, exhibiting a convergence effect.

## 4. Conclusions

This paper proposes an innovative adaptive learning rate method: a Lagrange interpolation-based black widow optimization algorithm developed to optimize the learning rate of the ResNet18 model, thereby significantly improving the training efficiency and performance of the model. The code developed in this paper is available at https://github.com/HJYJY/LIBWO (accessed on 30 May 2025).

To verify the effectiveness of LIBWONN, this paper selects nine new metaheuristic optimization algorithms (including DOA, ALA, SDO, CBSO, PSEQADE, COVIDOA, SASS, LSHADE-cnEpSin, AGSK) and conducts experiments on six public datasets covering various scenarios such as fashion, handwritten digits, and street-scene images. Additionally, 24 benchmark functions from CEC2017 and CEC2022 are chosen for the performance testing of the model. The experimental results demonstrate that LIBWONN performs excellently on multiple public datasets, thus significantly outperforming the original BWO algorithm without Lagrange interpolation.

By combining Lagrange interpolation techniques with the BWO algorithm, we effectively overcome the limitations of traditional learning rate optimization methods, especially in their convergence rate and generalization capability, which addresses the issue of BWO’s susceptibility to local optima. The LIBWONN algorithm dynamically constructs an interpolation function that can adaptively adjust the learning rate based on the training performance of the ResNet18 model. This mechanism enhances the robustness of the optimization process, which also effectively reduces the risk of oscillation and divergence caused by inappropriate learning rate choices during training.

Compared with traditional fixed learning rate methods and simple adaptive learning rate algorithms, LIBWONN can smoothly and effectively adjust the learning rate of the ResNet18 model, thereby accelerating convergence and improving final performance. This innovative approach provides new insights for training deep learning models that can significantly improve training results and overall model performance when handling complex tasks. Additionally, it offers a new solution for addressing the problem in which metaheuristic optimization algorithms suffer from local optima.

However, this method faces the issue of high computational costs, as each individual requires training and validation loss computation, which may significantly increase the training time when applied to deep neural networks and large-scale datasets. Moreover, LIBWONN may still suffer from local optima; although the pheromone mechanism guides the search, individuals may prematurely converge in complex high-dimensional search spaces, limiting the optimization performance and ultimately affecting the model’s learning capability and generalization ability.

Our current research mainly focuses on low-dimensional problems, but as optimization algorithms become more widely applied in practice, studying high-dimensional problems will become increasingly important. In the future, we plan to carry out more systematic and comprehensive experiments on high-dimensional tasks to evaluate the performance and efficiency of interpolation methods in complex environments, while also exploring potential optimization strategies to reduce computational costs.

Furthermore, considering practical deployment, future work will also explore how LIBWONN can be adapted for real-world applications such as embedded systems and real-time learning scenarios. These environments often demand lightweight models and rapid inference, which necessitates the development of simplified or accelerated versions of LIBWONN. Such efforts will help enhance the algorithm’s applicability in industrial and edge computing settings.

## Figures and Tables

**Table 1:** Comparison of results from neural network models.

Baseline	Training Loss	Test Loss	Training Accuracy	Test Accuracy	Precision	Recall	F1-Score
ResNet18	0.2610	0.3405	0.9946	0.9533	0.95	0.95	0.95
RNN	0.3721	0.3905	0.9703	0.9217	0.91	0.91	0.91
CNN	0.2895	0.3197	0.9857	0.9475	0.94	0.94	0.94
LSTM	0.3558	0.3729	0.9769	0.9328	0.93	0.93	0.93
GAN	0.4012	0.4156	0.9653	0.9105	0.90	0.90	0.90
VGG	0.2716	0.3652	0.9521	0.9549	0.92	0.92	0.92

**Table 2:** Comparison of results from interpolation methods.

Baseline	Training Loss	Test Loss	Training Accuracy	Test Accuracy	Precision	Recall	F1-Score
Newton	0.3102	0.3421	0.9862	0.9384	0.93	0.94	0.93
Spline	0.2905	0.3234	0.9901	0.9463	0.94	0.94	0.94
Quadratic	0.3257	0.3552	0.9837	0.9243	0.92	0.92	0.92
Linear	0.2998	0.3327	0.9874	0.9396	0.93	0.93	0.93
Chebyshev	0.3179	0.3481	0.9852	0.9358	0.93	0.93	0.93
Lagrange	0.2610	0.3405	0.9946	0.9533	0.95	0.95	0.95

**Table 3:** VHN dataset and comparison models.

Baseline	Training Loss	Test Loss	Training Accuracy	Test Accuracy	Precision	Recall	F1-Score	*p*-Value
LIBWONN	0.2717	0.2964	0.9628	0.9498	0.94	0.95	0.95	1.000
BWO	0.2867	0.3052	0.9391	0.9430	0.94	0.93	0.94	0.0176
DOA	0.3211	0.3194	0.9167	0.9361	0.93	0.93	0.93	0.0263
ALA	0.2713	0.3068	0.9197	0.9099	0.90	0.90	0.90	0.0381
SDO	0.2910	0.3201	0.9580	0.9398	0.93	0.94	0.93	0.0112
CBSO	0.3083	0.3230	0.9107	0.9243	0.92	0.92	0.92	0.2457
PSEQADE	0.3148	0.3169	0.9151	0.9241	0.93	0.93	0.93	0.1953
COVIDOA	0.2867	0.3052	0.9391	0.9430	0.91	0.93	0.94	0.0214
SASS	0.3007	0.3184	0.9376	0.9412	0.93	0.94	0.93	0.0226
LSHADE	0.3062	0.3215	0.9124	0.9268	0.92	0.92	0.92	0.2468
AGSK	0.3131	0.3152	0.9165	0.9237	0.93	0.93	0.93	0.0311
BOA	0.2648	0.3089	0.9423	0.9426	0.93	0.92	0.93	0.1429
WOA	0.3174	0.3146	0.9249	0.9312	0.93	0.93	0.93	0.2048

**Table 4:** MNIST dataset and comparison models.

Baseline	Training Loss	Test Loss	Training Accuracy	Test Accuracy	Precision	Recall	F1-Score	*p*-Value
LIBWONN	0.0994	0.0573	0.9912	0.9926	0.99	0.99	0.99	1.0000
BWO	0.1414	0.0721	0.9583	0.9776	0.98	0.98	0.98	0.0231
DOA	0.4732	0.0567	0.9873	0.9824	0.98	0.98	0.98	0.0442
ALA	0.1345	0.0872	0.9602	0.9729	0.97	0.97	0.97	0.0684
SDO	0.3221	0.0936	0.9898	0.9903	0.99	0.99	0.99	0.0012
CBSO	0.1384	0.1801	0.9322	0.9468	0.95	0.95	0.95	0.2350
PSEQADE	0.1142	0.1205	0.9558	0.9655	0.96	0.96	0.96	0.3120
COVIDOA	0.1164	0.1189	0.9524	0.9625	0.95	0.95	0.95	0.0291
SASS	0.1318	0.1768	0.9313	0.9458	0.95	0.95	0.95	0.3980
LSHADE	0.1117	0.1183	0.9548	0.9648	0.96	0.96	0.96	0.0094
AGSK	0.1152	0.1192	0.9515	0.9614	0.95	0.95	0.95	0.0761
BOA	0.1268	0.1017	0.9610	0.9702	0.97	0.97	0.97	0.2658
WOA	0.1195	0.0946	0.9635	0.9741	0.97	0.97	0.97	0.4682

**Table 5:** FASHION-MNIST dataset and comparison models.

Baseline	Training Loss	Test Loss	Training Accuracy	Test Accuracy	Precision	Recall	F1-Score	*p*-Value
LIBWONN	0.2610	0.3405	0.9946	0.9533	0.95	0.95	0.95	1.0000
BWO	0.3509	0.3741	0.9738	0.9332	0.93	0.93	0.93	0.0321
DOA	0.3981	0.4098	0.9755	0.9245	0.93	0.92	0.92	0.0154
ALA	0.3501	0.3714	0.9794	0.9272	0.93	0.93	0.93	0.0468
SDO	0.3191	0.3584	0.9760	0.9252	0.92	0.93	0.93	0.0023
CBSO	0.2853	0.3083	0.9612	0.9451	0.94	0.94	0.94	0.0214
PSEQADE	0.3271	0.3523	0.9742	0.9162	0.91	0.91	0.91	0.1832
COVIDOA	0.3496	0.3597	0.9649	0.9267	0.92	0.92	0.92	0.0587
SASS	0.2858	0.3088	0.9604	0.9449	0.94	0.94	0.94	0.0914
LSHADE	0.3289	0.3537	0.9724	0.9164	0.91	0.91	0.91	0.0009
AGSK	0.3493	0.3586	0.9652	0.9270	0.92	0.92	0.92	0.0275
BOA	0.3124	0.3367	0.9688	0.9341	0.93	0.93	0.93	0.2493
WOA	0.2988	0.3198	0.9705	0.9386	0.94	0.93	0.93	0.1955

**Table 6:** Intel Image Classification dataset and comparison models.

Baseline	Training Loss	Test Loss	Training Accuracy	Test Accuracy	Precision	Recall	F1-Score	*p*-Value
LIBWONN	0.2614	0.2862	0.9551	0.9410	0.95	0.93	0.93	1.0000
BWO	0.2604	0.3168	0.9478	0.9387	0.94	0.93	0.93	0.0453
DOA	0.3749	0.4537	0.9183	0.8984	0.90	0.90	0.90	0.0381
ALA	0.3846	0.4216	0.9372	0.9195	0.93	0.91	0.91	0.0624
SDO	0.3586	0.3870	0.9465	0.9301	0.94	0.92	0.92	0.0215
CBSO	0.3794	0.3989	0.9411	0.9314	0.94	0.92	0.92	0.0784
PSEQADE	0.3942	0.4367	0.9390	0.9307	0.93	0.92	0.92	0.1101
COVIDOA	0.3571	0.3498	0.9226	0.9354	0.92	0.92	0.92	0.0892
SASS	0.3796	0.3997	0.9430	0.9338	0.94	0.92	0.92	0.0247
LSHADE	0.3948	0.4295	0.9403	0.9267	0.93	0.92	0.92	0.0034
AGSK	0.3547	0.3479	0.9232	0.9347	0.92	0.92	0.92	0.0632
BOA	0.3205	0.3602	0.9401	0.9307	0.93	0.91	0.91	0.0547
WOA	0.3058	0.3417	0.9423	0.9332	0.93	0.92	0.92	0.0689

**Table 7:** RICE dataset and comparison models.

Baseline	Training Loss	Test Loss	Training Accuracy	Test Accuracy	Precision	Recall	F1-Score	*p*-Value
LIBWONN	0.0100	0.0133	0.9918	0.9951	0.99	0.99	0.99	1.0000
BWO	0.0309	0.0510	0.9967	0.9955	0.99	0.99	0.99	0.0224
DOA	0.0252	0.0703	0.9890	0.9872	0.98	0.98	0.98	0.0137
ALA	0.0378	0.0324	0.9816	0.9883	0.98	0.98	0.98	0.0459
SDO	0.1341	0.1452	0.9619	0.9769	0.97	0.97	0.97	0.0341
CBSO	0.1387	0.1208	0.9732	0.9754	0.97	0.97	0.97	0.0552
PSEQADE	0.1583	0.1378	0.9653	0.9582	0.96	0.96	0.96	0.0631
COVIDOA	0.1624	0.1289	0.9576	0.9597	0.95	0.95	0.95	0.0709
SASS	0.1413	0.1187	0.9730	0.9794	0.97	0.97	0.97	0.0508
LSHADE	0.1442	0.1339	0.9658	0.9502	0.96	0.96	0.96	0.0232
AGSK	0.1578	0.1315	0.9536	0.9591	0.95	0.95	0.95	0.0479
BOA	0.0853	0.0684	0.9784	0.9712	0.97	0.97	0.97	0.0385
WOA	0.0921	0.0726	0.9765	0.9698	0.96	0.96	0.96	0.0457

**Table 8:** CIFAR10 dataset and comparison models.

Baseline	Training Loss	Test Loss	Training Accuracy	Test Accuracy	Precision	Recall	F1-Score	*p*-Value
LIBWONN	0.0080	0.0917	0.9974	0.9307	0.93	0.93	0.93	1.0000
BWO	0.0030	0.0923	0.9967	0.9423	0.93	0.93	0.93	0.0214
DOA	0.0191	0.1459	0.9517	0.9094	0.91	0.91	0.91	0.0321
ALA	0.0257	0.0973	0.9295	0.9269	0.93	0.93	0.93	0.0457
SDO	0.0427	0.1072	0.9652	0.9184	0.92	0.92	0.92	0.0389
CBSO	0.1344	0.1242	0.9743	0.9760	0.97	0.97	0.97	0.0524
PSEQADE	0.1574	0.1495	0.9653	0.9503	0.96	0.96	0.96	0.0716
COVIDOA	0.1748	0.1271	0.9537	0.9583	0.95	0.95	0.95	0.0913
SASS	0.1461	0.1244	0.9682	0.9749	0.97	0.97	0.97	0.0782
LSHADE	0.1658	0.1363	0.9578	0.9482	0.96	0.96	0.96	0.0684
AGSK	0.1498	0.1303	0.9573	0.9520	0.95	0.95	0.95	0.0607
BOA	0.0652	0.1104	0.9637	0.9410	0.94	0.93	0.93	0.0493
WOA	0.0789	0.1187	0.9598	0.9357	0.93	0.92	0.92	0.0547

**Table 9:** CEC2017 testing functions.

Type	No.	Functions	Min
Unimodal Functions	1	Shifted and Rotated Bent Cigar Function	100
	2	Shifted and Rotated Sum of Different Power Function	200
	3	Shifted and Rotated Zakharov Function	300
Simple Multimodal Functions	4	Shifted and Rotated Rosenbrock’s Function	400
	7	Shifted and Rotated Lunacek Bi_Rastrigin’s Function	700
	8	Shifted and Rotated Non-Continuous Rastrigin’s Function	800
Hybrid Functions	13	Hybrid Function 3	1300
	14	Hybrid Function 4	1400
	15	Hybrid Function 5	1500
Composition Functions	21	Composition Function 1	2100
	22	Composition Function 2	2200
	23	Composition Function 3	2300

**Table 10:** CEC2022 testing functions.

Type	No.	Functions	Min
Unimodal Functions	1	Shifted and full Rotated Zakharov Function	300
Basic Functions	2	Shifted and full Rotated Rosenbrock’s Function	400
	3	Shifted and full Rotated Expanded Schaffer’s Function	600
	4	Shifted and full Rotated Non-Continuous Rastrigin’s Function	800
	5	Shifted and full Rotated Levy Function	900
Hybrid Functions	6	Hybrid Function 1	1800
	7	Hybrid Function 2	2000
	8	Hybrid Function 3	2200
	9	Composition Function 1	2300
Composition Functions	10	Composition Function 2	2400
	11	Composition Function 3	2600
	12	Composition Function 4	2700

**Table 11:** Comparison of results from 12 benchmark testing functions on CEC2017.

F		LIBWONN	BWO	DOA	ALA	SDO	CBSO	PSEQADE	COVIDOA	SASS	LSHADE	AGSK	BOA	WOA
F1	Ave	1.16 × 10^3^	1.09 × 10^3^	1.32 × 10^3^	1.24 × 10^3^	6.10 × 10^3^	4.30 × 10^3^	3.18 × 10^3^	1.75 × 10^3^	5.72 × 10^3^	4.10 × 10^3^	2.92 × 10^3^	7.50 × 10^3^	7.00 × 10^3^
	Std	6.79 × 10^3^	6.96 × 10^3^	8.05 × 10^3^	6.28 × 10^3^	3.55 × 10^3^	3.45 × 10^3^	6.82 × 10^3^	8.62 × 10^3^	4.59 × 10^3^	9.18 × 10^3^	1.08 × 104 × 10^4^	5.00 × 102 × 10^2^	4.20 × 102 × 10^2^
	Best	1.39 × 102 × 10^2^	3.72 × 102 × 10^2^	4.50 × 102 × 10^2^	3.88 × 102 × 10^2^	1.20 × 102 × 10^2^	1.39 × 102 × 10^2^	3.52 × 102 × 10^2^	2.10 × 102 × 10^2^	2.20 × 102 × 10^2^	5.68 × 102 × 10^2^	3.20 × 102 × 10^2^	8.00 × 10^3^	7.50 × 10^3^
F2	Ave	3.08 × 10^3^	4.41 × 10^3^	1.33 × 10^6^	7.75 × 10^6^	4.05 × 10^3^	3.55 × 10^3^	1.72 × 10^4^	4.90 × 10^3^	5.14 × 10^3^	2.54 × 10^4^	7.56 × 10^3^	1.20 × 10^4^	1.15 × 10^4^
	Std	1.03 × 10^4^	1.65 × 10^4^	3.75 × 10^7^	2.18 × 10^8^	1.38 × 10^4^	9.30 × 10^3^	1.24 × 10^4^	1.90 × 10^4^	1.38 × 10^4^	1.72 × 10^4^	2.52 × 10^4^	1.00 × 10^3^	9.00 × 10^2^
	Best	2.00 × 10^2^	2.00 × 10^2^	2.35 × 10^2^	2.17 × 10^2^	2.05 × 10^2^	2.02 × 10^2^	6.93 × 10^3^	2.02 × 10^2^	3.30 × 10^2^	1.04 × 10^4^	3.32 × 10^2^	6.00 × 10^2^	5.50 × 10^2^
F3	Ave	4.78 × 10^2^	4.51 × 10^2^	4.85 × 10^2^	1.49 × 10^2^	3.77 × 10^2^	4.25 × 10^2^	7.85 × 10^2^	4.58 × 10^3^	7.48 × 10^2^	1.45 × 10^3^	9.18 × 10^3^	8.00 × 10^2^	7.50 × 10^2^
	Std	5.01 × 10^2^	7.78 × 10^2^	1.02 × 10^3^	9.10 × 10^2^	5.05 × 10^2^	6.80 × 10^2^	6.15 × 10^2^	4.13 × 10^3^	1.03 × 10^3^	9.68 × 10^2^	6.82 × 10^3^	4.00 × 10^2^	3.80 × 10^2^
	Best	3.28 × 10^2^	3.04 × 10^2^	3.33 × 10^2^	3.05 × 10^2^	3.18 × 10^2^	3.16 × 10^2^	5.35 × 10^2^	3.72 × 10^2^	5.68 × 10^2^	9.81 × 10^2^	7.50 × 10^2^	2.20 × 10^3^	2.10 × 10^3^
F4	Ave	1.98 × 10^3^	2.12 × 10^3^	1.71 × 10^3^	1.88 × 10^3^	1.15 × 10^3^	1.28 × 10^3^	4.10 × 10^3^	5.26 × 10^3^	1.67 × 10^3^	5.80 × 10^3^	7.94 × 10^3^	6.50 × 10^3^	6.00 × 10^3^
	Std	4.23 × 10^3^	8.15 × 10^3^	5.90 × 10^3^	5.95 × 10^3^	4.35 × 10^3^	7.05 × 10^3^	7.05 × 10^3^	1.48 × 10^4^	1.08 × 10^4^	1.02 × 10^4^	2.03 × 10^4^	1.00 × 10^3^	9.00 × 10^2^
	Best	4.15 × 10^2^	4.22 × 10^2^	5.45 × 10^2^	6.20 × 10^2^	4.18 × 10^2^	4.55 × 10^2^	9.60 × 10^2^	6.10 × 10^2^	1.30 × 10^3^	2.59 × 10^3^	1.23 × 10^3^	7.01 × 10^2^	7.00 × 10^2^
F7	Ave	7.00 × 10^2^	7.01 × 10^2^	7.02 × 10^2^	7.02 × 10^2^	7.00 × 10^2^	7.00 × 10^2^	7.01 × 10^2^	7.02 × 10^2^	1.12 × 10^3^	1.13 × 10^3^	1.13 × 10^3^	1.10	1.10
	Std	1.14	1.45	1.11	1.22	6.80 × 10-	0.99	8.25 × 10^−1^	7.22 × 10^−1^	1.32	1.21	9.88 × 10^−1^	7.00 × 10^2^	7.00 × 10^2^
	Best	7.00 × 10^2^	7.00 × 10^2^	7.02 × 10^2^	7.02 × 10^2^	7.01 × 10^2^	7.01 × 10^2^	7.01 × 10^2^	7.02 × 10^2^	1.10 × 10^3^	1.10 × 10^3^	1.12 × 10^3^	8.10 × 10^2^	8.05 × 10^2^
F8	Ave	8.05 × 10^2^	8.07 × 10^2^	8.04 × 10^2^	8.05 × 10^2^	8.07 × 10^2^	8.03 × 10^2^	8.18 × 10^2^	8.16 × 10^2^	1.43 × 10^3^	1.50 × 10^3^	1.47 × 10^3^	1.20 × 10	1.15 × 10
	Std	1.02 × 10	1.32 × 10	1.04 × 10	1.21 × 10	8.80	6.75	1.44 × 10	6.05 × 10	1.25 × 10	2.92 × 10	1.17 × 10^2^	8.00 × 10^2^	8.00 × 10^2^
	Best	8.00 × 10^2^	8.00 × 10^2^	8.05 × 10^2^	8.06 × 10^2^	8.04 × 10^2^	8.05 × 10^2^	8.10 × 10^2^	8.14 × 10^2^	1.32 × 10^3^	1.35 × 10^3^	1.39 × 10^3^	1.32 × 10^3^	1.31 × 10^3^
F13	Ave	1.30 × 10^3^	1.31 × 10^3^	1.32 × 10^3^	1.36 × 10^3^	1.33 × 10^3^	1.33 × 10^3^	1.31 × 10^3^	1.32 × 10^3^	2.56 × 10^3^	2.41 × 10^3^	7.53 × 10^3^	1.20	1.15
	Std	1.14	1.45	1.11	1.20	6.78 × 10^−1^	0.97	8.30 × 10^−1^	5.28 × 10^−1^	1.45 × 10^3^	1.18 × 10^3^	1.74 × 10^3^	1.30 × 10^3^	1.30 × 10^3^
	Best	1.30 × 10^3^	1.30 × 10^3^	1.31 × 10^3^	1.30 × 10^3^	1.30 × 10^3^	1.30 × 10^3^	1.30 × 10^3^	1.31 × 10^3^	2.13 × 10^3^	2.45 × 10^3^	9.20 × 10^3^	8.10 × 10^2^	8.00 × 10^2^
F14	Ave	8.05 × 10^2^	8.07 × 10^2^	8.05 × 10^2^	8.07 × 10^2^	8.09 × 10^2^	8.03 × 10^2^	8.16 × 10^2^	8.15 × 10^2^	2.83 × 10^6^	1.15 × 10^7^	5.10 × 10^5^	1.15 × 10	1.10 × 10
	Std	1.02 × 10	1.32 × 10	1.05 × 10	1.23 × 10	8.80	6.72	1.47 × 10	6.05 × 10	1.02 × 10^8^	9.70 × 10^7^	1.12 × 10^6^	8.00 × 10^2^	8.00 × 10^2^
	Best	8.00 × 10^2^	8.00 × 10^2^	8.05 × 10^2^	8.07 × 10^2^	8.04 × 10^2^	8.07 × 10^2^	8.12 × 10^2^	8.17 × 10^2^	1.13 × 10^4^	5.52 × 10^4^	2.43 × 10^3^	3.00 × 10^3^	2.80 × 10^3^
F15	Ave	2.41 × 10^3^	3.30 × 10^3^	2.50 × 10^3^	2.40 × 10^3^	2.20 × 10^3^	2.08 × 10^3^	2.12 × 10^3^	6.10 × 10^3^	5.81 × 10^7^	5.65 × 10^8^	8.23 × 10^7^	8.00 × 10^2^	7.50 × 10^2^
	Std	7.50 × 10^2^	6.34 × 102	1.02 × 10^3^	1.08 × 10^3^	1.01 × 10^3^	1.06 × 10^3^	7.65 × 10^2^	9.45 × 10^2^	9.35 × 10^8^	1.91 × 10^9^	1.22 × 10^9^	1.50 × 10^3^	1.40 × 10^3^
	Best	2.19 × 10^3^	3.12 × 10^3^	1.77 × 10^3^	1.62 × 10^3^	1.55 × 10^3^	1.52 × 10^3^	1.64 × 10^3^	6.05 × 10^3^	1.04 × 10^4^	4.77 × 10^7^	2.68 × 10^6^	1.50 × 10^6^	1.40 × 10^6^
F21	Ave	1.50 × 10^5^	7.16 × 10^5^	1.17 × 10^7^	6.95 × 10^6^	9.75 × 10^5^	1.40 × 10^6^	5.90 × 10^6^	2.52 × 10^5^	5.39 × 10^7^	4.69 × 10^8^	1.02 × 10^8^	5.00 × 10^5^	4.80 × 10^5^
	Std	6.67 × 10^7^	1.80 × 10^7^	8.45 × 10^8^	3.92 × 10^8^	5.35 × 10^7^	8.40 × 10^7^	7.28 × 10^7^	8.88 × 10^5^	1.12 × 10^9^	2.35 × 10^9^	1.19 × 10^9^	1.20 × 10^5^	1.10 × 10^5^
	Best	1.32 × 10^3^	2.30 × 10^3^	1.68 × 10^3^	1.42 × 10^4^	1.02 × 10^3^	6.40 × 10^3^	3.35 × 10^4^	1.15 × 10^3^	3.51 × 10^3^	9.76 × 10^6^	1.47 × 10^5^	5.00 × 10^7^	4.80 × 10^7^
F22	Ave	3.79 × 10^7^	6.43 × 10^7^	1.23 × 10^8^	1.47 × 10^8^	4.35 × 10^7^	4.18 × 10^7^	4.60 × 10^8^	6.87 × 10^7^	5.72 × 10^3^	4.10 × 10^3^	2.92 × 10^3^	1.50 × 10^7^	1.40 × 10^7^
	Std	8.14 × 10^8^	8.77 × 10^8^	1.06 × 10^9^	1.34 × 10^9^	8.70 × 10^8^	8.50 × 10^8^	1.64 × 10^9^	8.85 × 10^8^	4.59 × 10^3^	9.18 × 10^3^	1.08 × 10^4^	1.00 × 10^6^	9.00 × 10^5^
	Best	2.70 × 10^3^	3.70 × 10^3^	5.15 × 10^4^	1.66 × 10^5^	1.59 × 10^4^	9.10 × 10^3^	2.45 × 10^7^	1.32 × 10^6^	2.20 × 10^2^	5.68 × 10^2^	3.20 × 10^2^	5.00 × 10^7^	4.90 × 10^7^
F23	Ave	3.38 × 10^7^	7.18 × 10^7^	1.41 × 10^8^	8.58 × 10^7^	9.30 × 10^7^	4.45 × 10^7^	4.00 × 10^8^	7.90 × 10^7^	5.14 × 10^3^	2.54 × 10^4^	7.56 × 10^3^	1.50 × 10^7^	1.40 × 10^7^
	Std	7.43 × 10^8^	1.13 × 10^9^	3.36 × 10^9^	1.62 × 10^9^	1.20 × 10^9^	8.30 × 10^8^	1.70 × 10^9^	8.40 × 10^8^	1.38 × 10^4^	1.72 × 10^4^	2.52 × 10^4^	1.00 × 10^6^	9.00 × 10^5^
	Best	3.87 × 10^3^	1.25 × 10^3^	4.40 × 10^4^	4.38 × 10^4^	1.18 × 10^4^	2.30 × 10^3^	6.50 × 10^6^	8.90 × 10^4^	3.30 × 10^2^	1.04 × 10^4^	3.32 × 10^2^	7.50 × 10^3^	7.00 × 10^3^
Rank		1	2	8	5	4	3	5	6	7	8	8	4	

**Table 12:** Comparison of results from 12 benchmark testing functions on CEC2022.

F		LIBWONN	BWO	DOA	ALA	SDO	CBSO	PSEQADE	COVIDOA	SASS	LSHADE	AGSK	BOA	WOA
F1	Ave	1.13 × 10^3^	1.92 × 10^3^	7.34 × 10^3^	2.12 × 10^6^	1.56 × 10^3^	2.56 × 10^3^	1.72 × 10^4^	3.11 × 10^3^	2.78 × 10^3^	1.92 × 10^4^	3.29 × 10^3^	1.64 × 10^3^	1.63 × 10^3^
	Std	1.03 × 10^3^	6.22 × 10^3^	1.15 × 10^5^	5.29 × 10^7^	5.12 × 10^3^	9.21 × 10^3^	1.15 × 10^4^	5.38 × 10^3^	8.56 × 10^3^	1.12 × 10^4^	5.11 × 10^3^	9.94 × 10^2^	1.08 × 10^3^
	Best	3.00 × 10^2^	3.00 × 10^2^	4.10 × 10^2^	4.25 × 10^2^	3.85 × 10^2^	3.90 × 10^2^	9.27 × 10^2^	6.00 × 10^2^	3.53 × 10^2^	9.76 × 10^2^	6.24 × 10^2^	6.00 × 10^2^	6.00 × 10^2^
F2	Ave	9.94 × 10^2^	1.08 × 10^3^	1.28 × 10^3^	2.42 × 10^3^	8.35 × 10^2^	1.02 × 10^3^	1.49 × 10^3^	8.64 × 10^2^	8.05 × 10^2^	1.50 × 10^3^	8.97 × 10^2^	8.03 × 10^2^	8.00 × 10^2^
	Std	1.06 × 10^3^	1.90 × 10^3^	5.18 × 10^3^	6.85 × 10^2^	9.64 × 10^2^	1.63 × 10^3^	1.14 × 10^3^	1.14 × 10^3^	1.35 × 10^3^	9.91 × 10^2^	9.91 × 10^2^	9.01 × 10^2^	9.07 × 10^2^
	Best	3.69 × 10^2^	4.50 × 10^2^	6.25 × 10^2^	6.22 × 10^2^	5.11 × 10^2^	5.33 × 10^2^	7.10 × 10^2^	4.91 × 10^2^	5.62 × 10^2^	7.32 × 10^2^	5.52 × 10^2^	1.10 × 10^3^	2.05 × 10^3^
F3	Ave	6.00 × 10^2^	6.00 × 10^2^	7.20 × 10^2^	7.15 × 10^2^	7.05 × 10^2^	7.08 × 10^2^	7.22 × 10^2^	7.10 × 10^2^	7.12 × 10^2^	7.24 × 10^2^	7.12 × 10^2^	2.87 × 10^3^	2.28 × 10^3^
	Std	7.93 × 10^−1^	7.02 × 10^−1^	5.10 × 10^−1^	5.56 × 10^−1^	6.08 × 10^−1^	5.35 × 10^−1^	5.99 × 10^−1^	6.42 × 10^−1^	6.00 × 10^−1^	6.83 × 10^−1^	7.42 × 10^−1^	2.21 × 10^10^	6.30 × 10^11^
	Best	6.00 × 10^2^	6.00 × 10^2^	7.12 × 10^2^	7.16 × 10^2^	7.18 × 10^2^	7.14 × 10^2^	7.19 × 10^2^	7.12 × 10^2^	7.12 × 10^2^	7.24 × 10^2^	7.12 × 10^2^	3.21 × 10^3^	3.14 × 10^3^
F4	Ave	8.03 × 10^2^	8.00 × 10^2^	9.19 × 10^2^	9.20 × 10^2^	9.22 × 10^2^	9.18 × 10^2^	9.35 × 10^2^	9.17 × 10^2^	9.45 × 10^2^	9.52 × 10^2^	9.50 × 10^2^	6.35 × 10^3^	4.82 × 10^3^
	Std	1.62	1.43	7.15 × 10^−1^	9.32 × 10^−1^	4.65 × 10^−1^	7.29 × 10^−1^	9.75 × 10^−1^	5.35 × 10^−1^	1.55	7.92 × 10	5.52 × 10	3.12 × 10^3^	4.33 × 10^3^
	Best	8.00 × 10^2^	8.01 × 10^2^	9.15 × 10^2^	9.17 × 10^2^	9.18 × 10^2^	9.14 × 10^2^	9.30 × 10^2^	9.16 × 10^2^	9.12 × 10^2^	9.13 × 10^2^	9.10 × 10^2^	3.05 × 10^3^	3.06 × 10^3^
F5	Ave	9.01 × 10^2^	9.07 × 10^2^	2.48 × 10^3^	1.87 × 10^3^	9.65 × 10^3^	1.56 × 10^3^	2.77 × 10^3^	3.72 × 10^3^	9.25 × 10^2^	9.42 × 10^2^	9.23 × 10^2^	1.13 × 10^3^	1.92 × 10^3^
	Std	1.21 × 10	1.07 × 10	3.02 × 10^3^	9.52 × 10^3^	5.85 × 10^3^	1.45 × 10^3^	6.82 × 10^3^	9.58 × 10^3^	7.23 × 10	9.75 × 10	5.12 × 10	9.94 × 10^2^	1.08 × 10^3^
	Best	9.00 × 10^2^	9.00 × 10^2^	2.70 × 10^4^	3.15 × 10^4^	2.35 × 10^3^	3.25 × 10^3^	3.10 × 10^5^	2.76 × 10^3^	9.14 × 10^2^	9.32 × 10^2^	9.19 × 10^2^	6.00 × 10^2^	6.00 × 10^2^
F6	Ave	1.10 × 10^3^	2.05 × 10^3^	3.47 × 10^3^	3.75 × 10^3^	3.28 × 10^3^	3.10 × 10^3^	5.10 × 10^3^	3.04 × 10^3^	1.68 × 10^3^	2.52 × 10^3^	3.62 × 10^3^	8.03 × 10^2^	8.00 × 10^2^
	Std	2.25 × 10^3^	8.93 × 10^3^	1.74 × 10^3^	7.20 × 10^3^	7.55 × 10^2^	1.75 × 10^3^	4.20 × 10^3^	6.20 × 10^2^	1.38 × 10^3^	6.92 × 10^3^	8.02 × 10^3^	9.01 × 10^2^	9.07 × 10^2^
	Best	1.90 × 10^3^	2.79 × 10^3^	2.56 × 10^3^	2.43 × 10^3^	2.32 × 10^3^	2.28 × 10^3^	2.50 × 10^3^	2.45 × 10^3^	3.12 × 10^3^	3.02 × 10^5^	2.52 × 10^3^	1.10 × 10^3^	2.05 × 10^3^
F7	Ave	2.87 × 10^3^	2.28 × 10^3^	4.89 × 10^14^	1.22 × 10^13^	2.78 × 10^13^	4.08 × 10^12^	1.08 × 10^11^	5.14 × 10^10^	2.92 × 10^3^	5.12 × 10^3^	2.85 × 10^3^	2.87 × 10^3^	2.28 × 10^3^
	Std	1.98 × 10^3^	1.32 × 10^3^	7.35 × 10^15^	1.75 × 10^14^	4.25 × 10^14^	5.13 × 10^13^	4.82 × 10^11^	5.24 × 10^12^	1.56 × 10^3^	4.70 × 10^3^	6.12 × 10^2^	2.21 × 10^10^	6.30 × 10^11^
	Best	2.04 × 10^3^	2.05 × 10^3^	3.52 × 10^3^	4.72 × 10^3^	3.45 × 10^3^	3.56 × 10^3^	1.05 × 10^6^	2.63 × 10^3^	2.58 × 10^3^	2.74 × 10^3^	2.62 × 10^3^	3.21 × 10^3^	3.14 × 10^3^
F8	Ave	2.21 × 101^0^	6.30 × 10^11^	3.80 × 10^3^	4.07 × 10^3^	3.28 × 10^3^	3.50 × 10^3^	4.78 × 10^3^	3.56 × 10^3^	4.02 × 10^13^	1.12 × 10^11^	5.22 × 10^11^	6.35 × 10^3^	4.82 × 10^3^
	Std	1.62 × 10^12^	8.64 × 10^12^	2.55 × 10^3^	4.75 × 10^3^	7.80 × 10^2^	9.48 × 10^2^	1.90 × 10^3^	1.12 × 10^3^	5.34 × 10^14^	5.11 × 10^12^	5.06 × 10^13^	3.12 × 10^3^	4.33 × 10^3^
	Best	2.84 × 10^3^	2.39 × 10^3^	3.18 × 10^3^	3.22 × 10^3^	3.06 × 10^3^	3.08 × 10^3^	3.61 × 10^3^	3.04 × 10^3^	3.70 × 10^3^	1.14 × 10^6^	2.72 × 10^3^	3.05 × 10^3^	3.06 × 10^3^
F9	Ave	3.21 × 10^3^	3.14 × 10^3^	4.10 × 10^3^	3.98 × 10^3^	3.61 × 10^3^	4.15 × 10^3^	4.55 × 10^3^	5.03 × 10^3^	3.56 × 10^3^	4.35 × 10^3^	3.88 × 10^3^	1.13 × 10^3^	1.92 × 10^3^
	Std	1.39 × 10^3^	1.01 × 10^3^	2.84 × 10^3^	2.92 × 10^3^	2.76 × 10^3^	2.60 × 10^3^	2.16 × 10^3^	4.38 × 10^2^	9.15 × 10^2^	1.77 × 10^3^	1.05 × 10^3^	9.94 × 10^2^	1.08 × 10^3^
	Best	2.30 × 10^3^	2.65 × 10^3^	3.36 × 10^3^	3.28 × 10^3^	3.10 × 10^3^	2.59 × 10^3^	3.24 × 10^3^	5.08 × 10^3^	2.78 × 10^3^	3.51 × 10^3^	2.94 × 10^3^	6.00 × 10^2^	6.00 × 10^2^
F10	Ave	6.35 × 10^3^	4.82 × 10^3^	4.42 × 10^3^	5.54 × 10^3^	4.55 × 10^3^	3.40 × 10^3^	4.62 × 10^3^	4.87 × 10^3^	4.12 × 10^3^	4.76 × 10^3^	5.12 × 10^3^	8.03 × 10^2^	8.00 × 10^2^
	Std	6.88 × 10^3^	2.24 × 10^3^	5.15 × 10^3^	2.21 × 10^4^	8.05 × 10^3^	2.02 × 10^3^	3.75 × 10^3^	4.32 × 10^3^	2.50 × 10^3^	2.10 × 10^3^	4.12 × 10^2^	9.01 × 10^2^	9.07 × 10^2^
	Best	2.44 × 10^2^	2.59 × 10^3^	2.95 × 10^3^	3.09 × 10^3^	3.01 × 10^3^	2.98 × 10^3^	3.40 × 10^3^	3.00 × 10^3^	2.67 × 10^3^	3.50 × 10^3^	5.11 × 10^3^	1.10 × 10^3^	2.05 × 10^3^
F11	Ave	3.12 × 10^3^	4.33 × 10^3^	3.56 × 10^3^	3.62 × 10^3^	3.49 × 10^3^	3.29 × 10^3^	3.90 × 10^3^	4.18 × 10^3^	3.56 × 10^3^	4.53 × 10^3^	4.82 × 10^3^	2.87 × 10^3^	2.28 × 10^3^
	Std	1.07 × 10^3^	4.89 × 10^3^	4.60 × 10^2^	5.82 × 10^2^	2.90 × 10^2^	2.68 × 10^2^	3.71 × 10^2^	8.24 × 10	1.90 × 10^3^	3.87 × 10^3^	4.01 × 10^3^	2.21 × 10^10^	6.30 × 10^11^
	Best	2.60 × 10^3^	2.61 × 10^3^	3.20 × 10^3^	3.25 × 10^3^	3.08 × 10^3^	3.15 × 10^3^	4.54 × 10^3^	4.27 × 10^3^	2.83 × 10^3^	3.23 × 10^3^	2.80 × 10^3^	3.21 × 10^3^	3.14 × 10^3^
F12	Ave	3.05 × 10^3^	3.06 × 10^3^	7.34 × 10^3^	2.12 × 10^6^	1.56 × 10^3^	2.56 × 10^3^	1.72 × 104	3.11 × 10^3^	3.25 × 10^3^	3.96 × 10^3^	4.12 × 10^3^	6.35 × 10^3^	4.82 × 10^3^
	Std	1.72 × 10^2^	2.07 × 10^2^	1.15 × 10^5^	5.29 × 10^7^	5.12 × 10^3^	9.21 × 10^3^	1.15 × 10^4^	5.38 × 10^3^	2.85 × 10^2^	4.00 × 10^2^	8.52 × 10	3.12 × 10^3^	4.33 × 10^3^
	Best	2.74 × 10^3^	2.94 × 10^3^	4.10 × 10^2^	4.25 × 10^2^	3.85 × 10^2^	3.90 × 10^2^	9.27 × 10^2^	6.00 × 10^2^	3.48 × 10^3^	4.56 × 10^3^	4.23 × 10^3^	3.05 × 10^3^	3.06 × 10^3^
Rank		1	2	4	4	5	3	7	7	6	7	7	6	6

**Table 13:** The results of the ablation study.

Baseline	Training Loss	Test Loss	Training Accuracy	Test Accuracy	Precision	Recall	F1-Score
LIBWONN	0.2610	0.3405	0.9946	0.9533	0.95	0.95	0.95
BWO	0.3509	0.3741	0.9738	0.9332	0.93	0.93	0.93
Fixed LR Optimization (Fixed LR)	0.3800	0.4200	0.9700	0.9200	0.91	0.91	0.91

**Table 14:** The results of the sensitivity analysis experiment.

Population	Iterations	Training Loss	Test Loss	Training Accuracy	Test Accuracy	Precision	Recall	F1-Score
10	50	0.3800	0.4200	0.9700	0.9200	0.91	0.91	0.91
30	50	0.3680	0.4090	0.9730	0.9250	0.92	0.92	0.92
50	50	0.3580	0.3960	0.9750	0.9300	0.92	0.92	0.92
100	50	0.3500	0.3800	0.9765	0.9330	0.93	0.93	0.93
10	100	0.3685	0.4145	0.9745	0.9220	0.91	0.91	0.91
30	100	0.3540	0.3980	0.9780	0.9260	0.93	0.92	0.92
50	100	0.3300	0.3700	0.9820	0.9340	0.94	0.93	0.94
100	100	0.3200	0.3600	0.9840	0.9450	0.94	0.94	0.94
10	200	0.3215	0.3920	0.9800	0.9280	0.92	0.92	0.92
30	200	0.3100	0.3810	0.9830	0.9370	0.93	0.93	0.93
50	200	0.2900	0.3530	0.9855	0.9445	0.94	0.94	0.94
100	200	0.2543	0.3078	0.9915	0.9553	0.95	0.95	0.95
150	200	0.2501	0.3050	0.9920	0.9560	0.95	0.95	0.95
10	300	0.3200	0.3900	0.9805	0.9295	0.92	0.92	0.92
30	300	0.3080	0.3795	0.9835	0.9380	0.93	0.93	0.93
50	300	0.2890	0.3525	0.9860	0.9450	0.94	0.94	0.94
100	300	0.2520	0.3050	0.9918	0.9555	0.95	0.95	0.95

## Data Availability

The source code used in this work can be retrieved from the following Github link: https://github.com/HJYJY/LIBWO (accessed on 30 May 2025).
